# Restricted HIV-1 diversity in people with HIV and exceptionally low viral reservoirs

**DOI:** 10.3389/fimmu.2026.1904455

**Published:** 2026-10-30

**Authors:** Irene González-Navarro, Ramon Lorenzo-Redondo, Cristina Gálvez, Victor Urrea, Lidia Garrido-Sanz, Javier Martinez-Picado, Maria Salgado

**Affiliations:** 1 IrsiCaixa, Badalona, Barcelona, Spain; 2 Department of Cellular Biology, Physiology and Immunology, Faculty of Medicine, Universitat Autònoma de Barcelona, Barcelona, Spain; 3 Department of Medicine, Division of Infectious Diseases, Feinberg School of Medicine, Northwestern University, Chicago, IL, United States; 4 Center for Pathogen Genomics and Microbial Evolution, Northwestern University Havey Institute for Global Health, Chicago, IL, United States; 5 CIBERINFEC, Carlos III Health Institute, Madrid, Spain; 6 University of Vic – Central University of Catalonia (UVic-UCC), Vic, Spain; 7 Catalan Institution for Research and Advanced Studies (ICREA), Barcelona, Spain; 8 Germans Trias i Pujol Research Institute (IGTP), Badalona, Spain

**Keywords:** entropy, evolution, HIV, LoViReT, reservoir

## Abstract

**Background:**

HIV-1 cure strategies aim to eradicate viral reservoirs that persist despite effective antiretroviral therapy (ART). Low Viral Reservoir Treated (LoViReT) individuals—characterized by exceptionally low proviral DNA levels during ART—offer a unique model to investigate mechanisms underlying reservoir restriction. This study characterizes the genetic features of HIV-1 populations within blood-derived reservoirs of LoViReT individuals.

**Methods:**

We sequenced regions of *gag*, *pol*, and *env* genes from 19 LoViReTs and 21 matched ART-treated controls, defined by proviral DNA levels of <100 and >100 HIV-1-DNA copies/10^6^ CD4^+^ T-cells, respectively. Paired pre-ART samples from 7 LoViReTs and 8 controls were also analyzed. Genetic diversity was assessed using Shannon entropy and intra-quasispecies weighted genetic distance. Phylogenetic analyses and statistical modeling were performed to compare viral populations and explore viral-host associations between groups.

**Findings:**

LoViReT individuals exhibited significantly lower intra-host Shannon entropy under ART than controls, with *env* gene contributing most to this difference. Reconstructed quasispecies haplotypes confirmed reduced viral diversity in LoViReT participants, particularly in *env*. A subset of LoViReTs also showed increased APOBEC-mediated hypermutation in *pol*, suggesting greater accumulation of deleterious mutations. Matched pre- and post-ART sequences indicated a similar trend toward reduced diversity before treatment initiation in LoViReTs with increased genetic frequencies of matched HLA-restricted epitopes.

**Interpretation:**

These findings indicate that LoViReT individuals maintain a less diverse viral reservoir, potentially reflecting differential immune patterns before therapy and explaining their reduced reservoir size during ART.

## Introduction

Although antiretroviral therapy (ART) effectively suppresses viremia in people living with HIV-1 (PWH), it does not eradicate the latent reservoir, which remains silently integrated mainly within resting CD4^+^ T-cells (1). This reservoir retains the minimal genetic diversity required for viral rebound following treatment interruption, thereby preserving HIV-1 fitness. Such diversity arises from the virus’s high mutation and recombination rates, coupled with prolific progeny production (2). Consequently, intra-host HIV-1 populations exist as heterogeneous spectra of mutant genomes, referred to as quasispecies. This extensive genetic diversity, along with the rapid evolutionary capacity of HIV-1, poses significant challenges to both natural immune control (e.g., by neutralizing antibodies or T-cell responses) and the efficacy of antiretroviral agents (3). These factors remain major barriers to achieving sustained viral control in the absence of treatment and underscore the urgent need for effective curative strategies that target the HIV-1 reservoir, with particular consideration of its extensive genetic diversity (2). However, the precise role of this variability in reservoir reduction remains unclear, complicating the standardization, effectiveness, and universal applicability of HIV-1 eradication approaches.

The size, composition, and dynamics of the viral reservoir vary among individuals. Reduced reservoir size in Elite Controllers (ECs) has been related to markedly lower plasma viral diversity and more robust immune responses that effectively constrain replication despite residual viral evolution (4). ECs also show restricted viral evolution within peripheral blood mononuclear cells (PBMCs), with the majority of viruses being homogeneous and originating from ancestral variants through clonal expansion (5). Although ECs possess unique traits that allow them to control HIV-1 infection, studies involving individuals who initiate treatment early have revealed similar patterns, including smaller reservoirs and reduced diversity within their viral quasispecies (6, 7). These findings suggest that limited viral diversity and evolutionary capacity can occur not only in ECs but also in other PWH under specific immune pressures or treatment conditions.

Our group has previously defined Low Viral Reservoir Treated (LoViReT) individuals as a subset of PWH characterized by extremely low proviral DNA levels under ART, initiated either during the acute or chronic phase of infection (<50 HIV-1-DNA copies/10^6^ PBMCs; <100 HIV-1-DNA copies/10^6^ CD4^+^ T-cells) (8). Earlier work has shown that LoViReTs may present detectable viral loads (VL) (approaching 10^5^ HIV-1-RNA copies/mL) but maintain distinctly lower reservoir levels—even before ART—compared with controls (9). We have partially attributed these reduced reservoir sizes to clinical factors such as earlier treatment initiation, higher nadir CD4^+^ T-cell counts, and the use of integrase strand transfer inhibitors (InSTIs) in initial ART regimens (10). Nonetheless, we anticipate that additional, yet unidentified immunovirological factors contribute to the unique LoViReT phenotype and its characteristic reservoir profile. To our knowledge, no studies have directly investigated the impact of viral diversity on reservoir size outside of ECs or early-treated individuals. We hypothesize that the reduced reservoir observed in LoViReTs may be linked to constrained viral diversity and limited evolutionary dynamics—even prior to ART initiation—which could facilitate viral eradication.

To address this, we conducted a longitudinal analysis to evaluate the viral diversity and evolutionary dynamics of reservoirs before and after ART in a previously characterized subgroup of LoViReT individuals, comparing them with matched controls with higher reservoir levels (8). Our goal is to understand how viral diversity affects the establishment and maintenance of the HIV-1 reservoir, serving as an indirect indicator of pre-ART immune pressure and viral replication. This approach may help identify novel targets to enhance reservoir elimination and thereby advance efforts toward viral eradication.

## Methods

### Study participants

Based on continued consent and sample availability, 19 LoViReT participants (both early and chronically treated) under regular follow-up at Hospital Germans Trias i Pujol and Hospital Clinic (Barcelona, Spain) were included and matched to 21 controls based on 17 clinical variables using a previously described random forest–based approach to identify participants with the greatest overall similarity (8). All the individuals fulfilled the inclusion criterion, which involved being on suppressive ART with undetectable viremia (<50 HIV-1 RNA copies/mL plasma) for at least 3 years and exhibiting proviral levels below or above 100 HIV-1 DNA copies/10^6^ CD4^+^ T cells in LoViReTs and controls, respectively (8). Blood samples were collected on ART following a median of 14 years of viral suppression [IQR, 9–22]. In 7/19 LoViReTs and 8/21 controls, matched pre-ART samples were available for comparison. All participants in the study provided signed informed consent. The study was approved by the Ethics Committee at both recruiting centers (reference number: PI-14-083).

### Sex as a biological variable

Our study examined both male and female participants. Sex at birth was self-reported and used as a biological variable in analyses.

### Total HIV-1-DNA quantification

We evaluate the size of the viral reservoir by measuring total HIV-1-DNA in purified CD4^+^ T-cell lysed extracts (at both pre- and post-ART samples) through droplet digital polymerase chain reaction (ddPCR, BioRad), as previously reported (11). Briefly, 5’*LTR* and *gag* regions were amplified, while the *RPP30* housekeeping gene was measured in parallel to normalize sample input. Raw ddPCR data were analyzed using the QX100™ Droplet Reader and QuantaSoft v.1.6 software (Bio-Rad, USA). Proviral quantification for each individual corresponded to the highest value obtained from the 2 primer sets used (either 5’ *LTR* or *gag*).

### Viral tropism and subtyping

Viral coreceptor tropism and subtype were assessed on proviral DNA by sequencing HIV-1 *gp120* hypervariable region 3 (V3) and subsequently predicted through the Geno2Pheno algorithm (GENAFOR) (12). Tropism was inferred using the Genotypic Prediction of Coreceptor Usage tool with a false-positive rate (FPR) of 10% (non-R5 tropism: FPR ≤10%), whereas the HIV-1 subtype was estimated using the Geno2Pheno Virus Detection and Subtyping Tool. The primers used for these analyses were distinct from those used here for amplification of the env region analyzed for viral diversity and are therefore not included in **Supplementary Table 2**.

### Viral genome sequencing

We amplified and deep-sequenced regions of the HIV-1 structural genes *gag*, *pol*, and *env* from viral populations in blood reservoirs. Genomic DNA was extracted from purified CD4^+^ T-cells (pre-ART mean 1.8M cells; post-ART mean 3.6M cells) isolated from peripheral blood mononuclear cells (PBMCs) and used for nested PCR. Bulk amplified products were subsequently used for Illumina MiSeq sequencing to study the viral genetic diversity of the virus. The successful amplifications are summarized in **Supplementary Table 1**.

In brief, two primer sets per gene were used to target specific regions (~280–550 bp; **Supplementary Figure 1** and **Supplementary Table 2**) of the HIV-1 genes by a two-step nested PCR. After purification, samples were tagged using Primer-ID sequences to identify each original template copy prior to Nextera XT DNA library preparation, pooled, and sequenced on an Illumina MiSeq platform using the MiSeq Reagent Micro Kit v2 (300-cycles), with an average depth of 91,609.97 paired-end reads per sample.

### HLA typing

High‐resolution HLA typing of class I (A, B, Cw) and class II (DRB1, DQA1, DQB1, DPB1) loci was performed using sequence‐based methods to identify protective (B*57, Cw*06, B*27, Cw*01, A*25, B*52) and risk alleles (Cw*04, B*35, A*23, A*29, B*55, B*49) (13).

### Intra-host diversity assessment by Shannon entropy

To study and compare intra-host diversification in different participants, Shannon entropy was estimated from nucleotide frequencies at each position using the iVar (14) *variants* function with a frequency threshold >1% from deep sequencing reads. Paired-end reads were quality-trimmed using Trimmomatic v0.3 (15) in paired-end mode using the standard Illumina paired-end trimming parameters recommended in the Trimmomatic manual: leading and trailing bases with a Phred quality score below 3 were removed to strip unreliable end bases, followed by sliding-window trimming (4 bp window, mean quality threshold 15) as the primary quality filter. Reads shorter than 36 bp after trimming were discarded. Shannon entropy was subsequently calculated as: Sh=−∑(pi)· log2(pi); where *Sh* represents the Shannon entropy at each position and *pi* the frequency of each nucleotide at that position. Shannon entropy calculations were limited to positions with a minimum read depth of 1,000 reads in every sample analyzed to ensure the robustness of the diversity estimation. Shannon entropy estimates were not normalized according to the number of original template molecules.

Significant differences in overall *Sh* entropy between controls and LoViReTs, both pre- and post-ART, were also assessed in subjects with matched samples at both time points for at least one gene by summing the genetic entropy values for each nucleotide position in every sample. We also tested changes in entropy per gene by adding entropy at each genetic position.

### Quasispecies weighted genetic distance calculation

We also performed a reference-based assembly of the sequencing reads using the HAPHPIPE v1.0.3 protocol (16) and HXB2 reference genome (17). Reads were error-corrected using SPAdes v3.13.1 (18). With the assembled reads, we performed probabilistic inference of intra-host viral quasispecies using QuasiRecomb v1.2 (19). Reconstructed haplotypes were then used to calculate intra-host diversity using pairwise distances weighted by the estimated frequency of the haplotype with an in-house python script that utilizes the *DistanceCalculator* function in Biopython v1.79 (20).

### APOBEC-mediated hypermutation frequency evaluation

Finally, we assessed APOBEC-driven hypermutation in each haplotype reconstructed through the HYPERMUT 3.0 tool from the Los Alamos National Laboratory (LANL) HIV Immunology Database (21). The analysis included all possible APOBEC motifs (mainly G and F). Haplotypes with a *p* < 0.1 in the Fisher’s exact test performed by the tool were considered hypermutants. Hypermutant frequencies were then assessed within the quasispecies of each sample.

### Phylogenetic analysis

All sequences from the inferred haplotypes were aligned using MAFFT v7.453 (22, 23) software including the 2021 Consensus B reference genome (24). Regions with poor alignment scores were eliminated using the Gblocks (25, 26) tool implemented in *seaview* v5.0.4 (27). Maximum-likelihood phylogenies were inferred with IQ-Tree v2.0.5 (28) using its *Model Finder* function before each analysis to estimate the nucleotide substitution model best-fitted for each dataset by means of Bayesian information criterion (BIC). We assessed the tree topology for each phylogeny with the Shimodaira–Hasegawa approximate likelihood-ratio test (SH-aLRT) with 1,000 replicates (29).

### Statistical modeling and testing

All statistical modeling analyses were conducted in R v4.4.1 (30). Simple group comparisons were performed using Wilcoxon rank sum and signed-rank tests. To assess differences in post-ART within-host diversity or hypermutation levels between groups, linear regression models were fitted with the *lme4 (*31) package v1.1–37 using either log_10_-transformed overall *Sh*, *Sh* per region, hypermutation frequency per region, or quasispecies pairwise weighted genetic distance as the outcome variables in each model. Time from HIV-1 diagnosis to sampling was log_10_-transformed and included as a continuous covariate in every model to account for differences in the duration of viral evolution up to the time of sampling, while participant group served as a categorical predictor. When considering pre- and post-ART samples, linear mixed-effects models were fitted to account for repeated measurements within individuals and to evaluate group- and treatment-specific effects, using log_10_-transformed overall *Sh*, genetic region *Sh*, or quasispecies genetic distance as outcomes. Time from HIV-1 diagnosis to sampling was log_10_-transformed and included as a continuous covariate, while ART status, participant group, and their interaction were modeled as fixed effects to test group-specific differences across treatment conditions. Individual identity was modeled as a random intercept to account for within-subject correlation arising from longitudinal or per-site measurements. For every model, estimated marginal means were computed for the group variable using the *emmeans (*32) package v2.0.0 to assess group-level differences while adjusting for time from HIV-1 diagnosis to sampling. Pairwise group comparisons were performed with false discovery rate (FDR) correction to account for multiple testing in analyses stratified by regions of the three genes (*gag*, *pol*, and *env*) and in paired comparisons between pre- and post-ART samples.

Post-ART samples were analyzed for associations of overall *Sh* with clinical and demographic variables. Categorical variables with empty strings were converted to missing values and unused factor levels were removed. To balance model complexity with data completeness, we implemented a variable-selection procedure that maximized the number of covariates retained while enforcing a minimum proportion of complete observations. For each dataset, variables were ranked by increasing missingness, and an iterative trade-off curve was constructed to identify the largest set of predictors that preserved ≥90% complete cases. Only variables meeting these completeness thresholds were retained for downstream modeling. Prior to modeling, variables not biologically relevant to inference (e.g., identifiers, dates, and redundant timing variables) were excluded. Predictors exhibiting near-zero variance were also filtered using the *caret* R package (33) v7.0-1. Continuous predictors were centered and scaled, while the outcome variable (overall viral *Sh*) was log_10_-transformed to satisfy linear model assumptions. Initial full models included all retained predictors and were subjected to bidirectional stepwise selection based on Akaike’s information criterion (AIC). Model coefficients, global *p*-values, and confidence intervals were summarized using *gtsummary* (34) v2.4.0. Type III analysis of variance was performed to assess overall predictor significance. Estimated marginal means and pairwise contrasts for categorical predictors were computed using *emmeans* (32) v2.0.0, with FDR correction applied to multiple comparisons. The same approach was followed to analyze the associations with *pol* hypermutation levels.

### Multivariate dimensionality reduction and clustering analysis

For post-ART multivariate dimensionality reduction and clustering, a fully numeric metadata matrix was constructed to enable multivariate analysis. Ordinal clinical labels were converted to numeric variables, with the presence of HLA protective alleles encoded as +1, susceptible as −1, and the rest encoded as 0. To ensure complete-case multivariate modeling, a variable selection procedure was applied to maximize the number of retained variables while enforcing 100% sample completeness. Variables associated with identifiers, time intervals, composite clinical scores, label encodings, or redundant clinical measures were excluded. Additional biologically non-informative variables (including age at diagnosis, frailty index, and estimated time to infection) were removed prior to analysis. Principal component analysis (PCA) was performed on the filtered numeric dataset using the *FactoMineR* package v2.12, with all variables centered and scaled to unit variance. The number of retained components was determined empirically, and the first four principal components were examined. Scree plots, variable contribution plots, and biplots were used to assess variance explained and the relative contributions of individual variables to each component. PCA individual and biplot visualizations were further annotated by group, with confidence ellipses to assess separation between groups in reduced dimensional space.

### Analysis of HLA-specific anchor residue motifs in paired HIV-1 gag sequences

Paired pre- and post-ART HIV-1 *gag* sequences from a subset of participants with matched samples (5 LoViReTs and 3 controls) were analyzed to identify potential HLA anchor residue motifs using the HIV HLA Anchor Residue Motifs (Motif Scan) tool from LANL HIV Immunology Database (35). Reconstructed haplotype *gag* sequences per sample—excluding APOBEC hypermutants—were screened according to each participant’s HLA genotype using the default Motif Scan parameters (motif libraries: Marsh2000, SYFPEITHI, and other curated sources; motif length = 9 amino acids), enabling the detection of anchor residue motifs compatible with their specific HLA class I molecules. Positions within *gag* matching known HLA anchor residue patterns were identified, and motif frequencies were calculated for each sequence using the haplotype’s intra-quasispecies frequency. Frequencies were then compared between paired pre- and post-ART sequences for each participant. To statistically assess differences in motif frequencies while accounting for repeated measurements and motif-specific variability, we fitted a generalized linear mixed-effects regression model (GLMER) using *lme4*, with a Tweedie distribution (*statmod* v.1.5.1 R package) to better accommodate zero-inflated frequency data. The model specified motif frequency as the outcome and included ART status (pre- vs post-ART), patient group, and their interaction (ART × Group) as fixed effects, with years since infection included as a continuous covariate to account for viral diversification over time. Random intercepts for motif and patient were incorporated to account for motif-specific baseline variation and within-patient correlation across sequences. This approach enabled evaluation of ART-associated changes in HLA anchor residue motif frequencies across individuals while controlling for infection duration and hierarchical structure of the data. Statistical significance was evaluated at α = 0.05 after FDR adjustment.

### Data availability

Study data are not publicly available to preserve the privacy of research participants. Data can be made available by the corresponding author to any interested researcher following approval of a concept sheet summarizing the analysis to be performed. Deidentified participant data and a data dictionary can be made available and shared under a data transfer agreement. Requests for access to the LoViReT study data should be sent to msalgado@irsicaixa.es or jmpicado@irsicaixa.es.

## Results

### Study population characteristics

We selected 19 LoViReTs and 21 controls from a previously described cohort of PWH under suppressive ART for more than 3 years with known reservoirs (8–10). No statistically significant differences were observed between groups regarding clinical characteristics (**Table 1**). Both groups included early- and chronically treated participants, with LoViReTs showing a higher proportion of early-treated individuals (47%) than controls (33%), although this difference was not statistically significant (*p* = 0.63).

**Table 1:** Clinical characteristics of the study participants.

Variable	Abbreviation	Total (n = 40)	LoViReT group (n = 19)	Control group (n = 21)	*p*-value
Total HIV-1-DNA post-ART (copies/10^6^ CD4^+^ T-cells), median (min. **–** max.)^a^	Proviral	120 (2.6 – 1298.8)	25 (2.6 – 86.3)	340 (106.7 – 1298.8)	<0.0001*
Age at HIV-1 diagnosis (years), median [IQR]^a^	Age at diagnosis	34 [30 – 37]	35 [31 – 40]	33 [30 – 37]	0.42
Age at sampling (years), median [IQR]^a^	Age	51 [47 – 58]	51 [47 – 55]	53 [49 – 59]	0.52
Sex, *n* (%)^b^	Sex				1.00
Female		4 (10)	2 (11)	2 (10)	
Male		36 (90)	17 (89)	19 (90)	
HIV-1 subtype, *n* (%)^b,c^	Subtype				0.55
B		30 (75)	11 (58)	19 (90)	
Non-B		3 (8)	2 (11)	1 (5)	
Missing		7 (17)	6 (31)	1 (5)	
Tropism, *n* (%)^b,c^	Tropism				1.00
R5-tropic virus (CCR5)		28 (70)	11 (58)	17 (81)	
Non-R5-tropic virus (CXCR4 or dual-tropic)		4 (10)	1 (5)	3 (14)	
Missing		8 (20)	7 (37)	1 (5)	
Race, *n* (%)^b,c^	Race				0.13
Caucasian		30 (75)	14 (74)	16 (76)	
Non-caucasian		4 (10)	-	4 (19)	
Missing		6 (15)	5 (26)	1 (5)	
Route of HIV-1 transmission, *n* (%)^b^	RoT				1.00
MSM		21 (53)	10 (53)	11 (52)	
Non-MSM		14 (35)	6 (32)	8 (38)	
Missing		5 (13)	3 (16)	2 (10)	
Time from HIV-1 diagnosis to sampling (years), median [IQR]^a^	timeDtoS	17 [11 – 25]	15 [9 – 22]	20 [12 – 26]	0.44
Time from HIV-1 diagnosis to initiation of ART (years), median [IQR]^a^	timeDtoART	0.7 [0.2 – 3]	0.7 [0.1 – 5]	0.7 [0.3 – 2]	0.63
Early-treated, *n* (%)		16 (40)	9 (47)	7 (33)	
Chronically treated, *n* (%)		24 (60)	10 (53)	14 (67)	
Time from initiation of ART to sampling (years), median [IQR]^a^	timeARTtoS	14 [9 – 22]	12 [9 – 19]	17 [11 – 24]	0.28
Time from first undetectable VL to sampling (years), median [IQR]^a^	timeUtoS	13 [8.5 – 19.8]	11 [8 – 17]	14 [10 – 20]	0.23
Time from HIV-1 diagnosis to the first undetectable VL (years), median [IQR] ^a^	timeDtoU	1.2 [0.4 – 7]	0.9 [0.3 – 7]	3.5 [0.9 – 7]	0.24
Time from last detectable VL to sampling (years), median [IQR]^a^	timeLVtoS	11 [8 – 13]	11 [8 – 13]	11 [9 – 13]	0.56
Zenith VL (log_10_ copies/mL plasma), median [IQR]^a^	Zenith VL	4.7 [4.6 – 5.2]	4.7 [4.3 – 5.0]	4.7 [4.6 – 5.2]	0.46
Missing zenith data, *n* (%)		17 (43)	7 (37)	10 (48)	
CD4^+^ T-cell count	minCD4				0.23
Nadir (cells/µL), median [IQR]^a^		353 [298 – 445]	392 [344 – 437]	332 [280 – 449]	
Missing nadir data, *n* (%)		9 (23)	5 (26)	4 (19)	

Demographic, clinical, and virological parameters of LoViReT (n=19) and control (n=21) participants at study inclusion. Data are presented as median [IQR] or n (%).

### LoViReTs proviruses demonstrated lower entropy levels, especially in the env gene

We successfully amplified *gag*, *pol* and *env* gene regions in most participants despite the challenge posed by the low proviral burden in LoViReT samples (**Supplementary Table 1**). Intra-host viral diversity on ART was assessed through *Sh* estimation, which captured sequence variability based on nucleotide frequencies at each position. When calculating *Sh* across the three compiled regions, LoViReTs showed significantly lower diversity than controls (*p* = 0.003, **Figure 1A**) after fitting a linear regression model controlling for time since infection. Gene-specific analyses using the same approach while correcting for multiple comparisons across the three regions demonstrated similar patterns, with the *env* region accounting for most of the observed differences; however, this did not reach statistical significance (*p*-adj. = 0.07) (**Figure 1B**).

**Figure 1:**
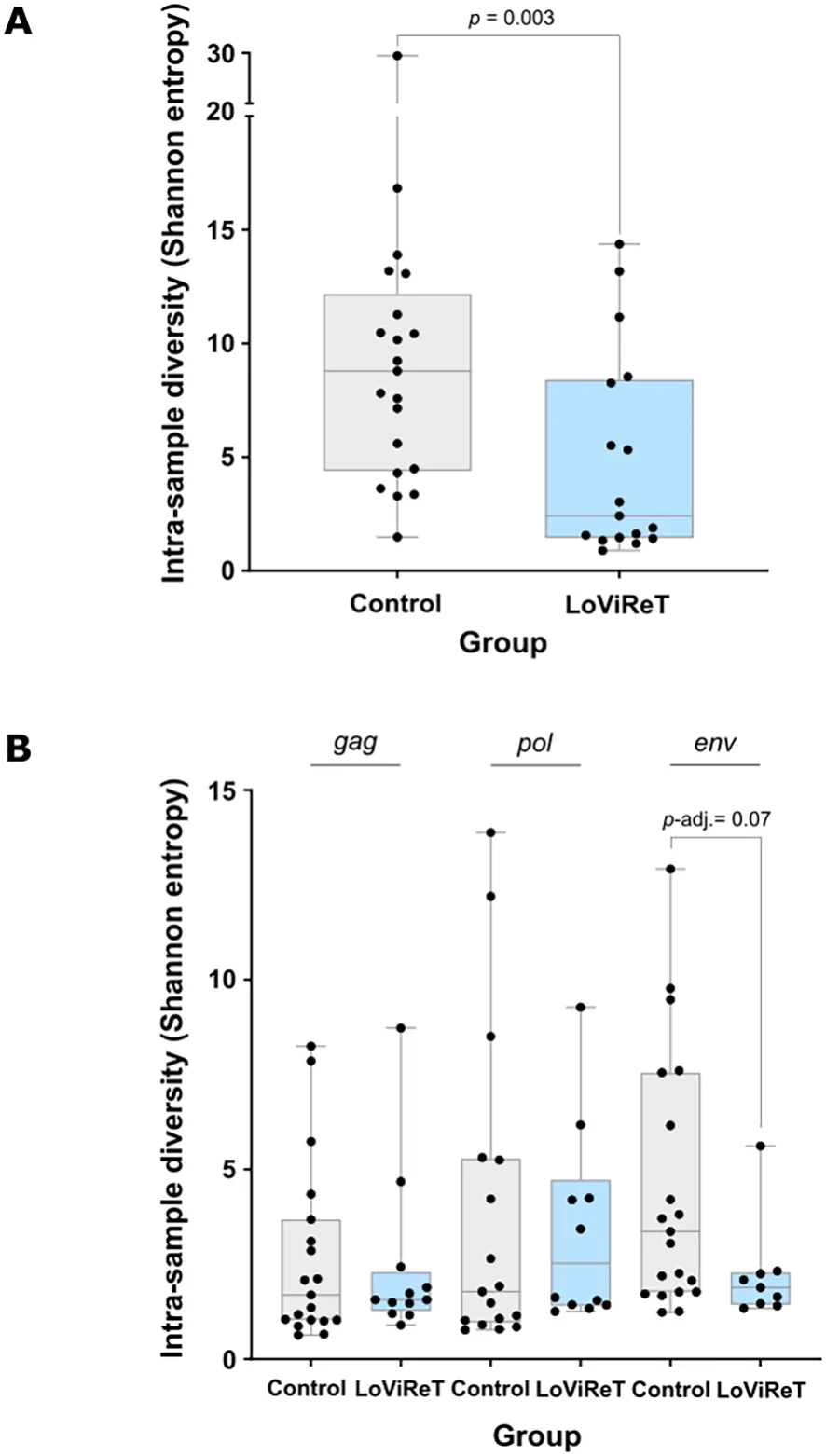
Intra-sample HIV-1 diversity. **(A)** Shannon entropy values per group. **(B)** Shannon entropy by gene region (*gag*, *pol*, *env*). LoViReTs (<100 HIV-1-DNA copies/10^6^ CD4^+^ T-cells; blue) show lower diversity in *env* compared with controls (>100 HIV-1-DNA copies/10^6^ CD4^+^ T-cells; gray).

### Haplotype variants indicated higher frequency of unique clonal sequences and lower diversity in LoViReTs

To analyze molecular evolution, we performed reference-guided mapping of sequencing reads, followed by viral quasispecies reconstruction. Studying the reconstructed viral haplotypes for each participant during ART allowed us to examine intra-host viral population dynamics within their reservoirs. Maximum-likelihood trees were inferred for the quasispecies in each amplified region to assess evolutionary relationships and HIV-1 population distributions. Consistent with *Sh* results, controls exhibited higher diversity, whereas LoViReTs had more homogeneous reservoirs largely dominated by a single haplotype. This difference was most pronounced in the *env* gene (**Figure 2A**), relative to *gag* and *pol* (**Supplementary Figures 2A, B**), as LoViReTs harbored a very limited number of distinct haplotypes, with the most prevalent one consistently occurring at high frequency; however, the observed difference was not statistically significant (*p*-adj. = 0.08).

**Figure 2:**
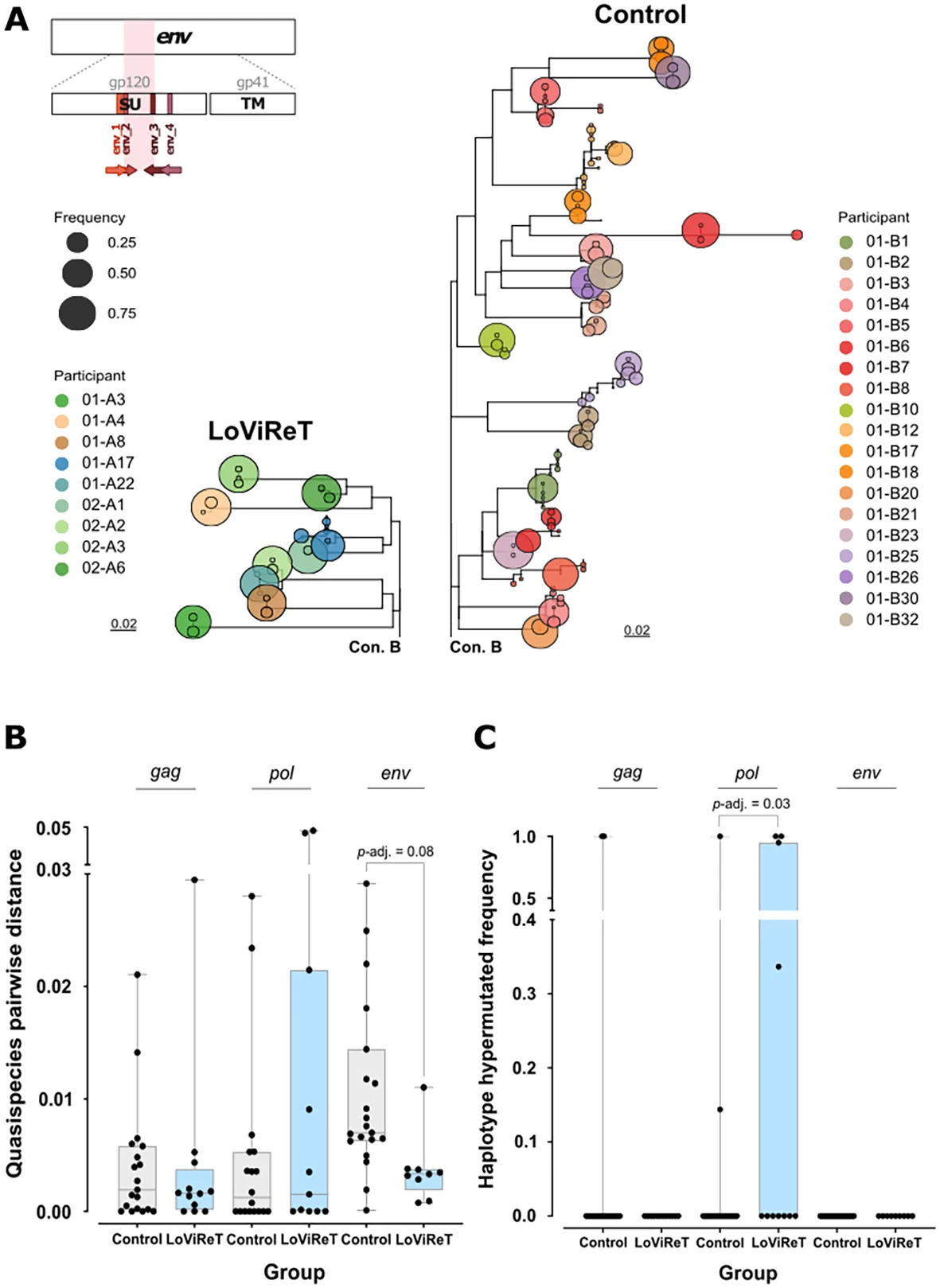
HIV-1 haplotype phylogenies, weighted quasispecies diversity, and APOBEC-driven hypermutation frequencies. **(A)** Maximum-likelihood tree of the *env* gene quasispecies. Tips represent unique haplotypes, sized by frequency, and color-coded by participant and group. **(B)** Genetic diversity per region (*gag*, *pol*, *env*). LoViReTs show lower diversity in *env* compared with controls. **(C)** APOBEC-driven hypermutation frequencies depicted per haplotype across gene regions, with most values clustered around a median of 0. LoViReTs (<100 HIV-1-DNA copies/10^6^ CD4^+^ T-cells) are highlighted in blue, while controls (>100 HIV-1-DNA copies/10^6^ CD4^+^ T-cells) are colored in light gray. Con. B, Consensus B.

We next estimated quasispecies diversity as the average pairwise genetic distance of post-ART haplotypes weighted mean by their relative frequencies. In line with the phylogenetic patterns, single-gene analysis identified *env* as the main contributor to this variation, with LoViReT proviruses showing lower diversity than controls (LoViReTs=3.7x10–^3^ vs. controls=1.0x10^-2^; *p*-adj. = 0.08) (**Figure 2B**). Notably, *pol* was the only locus in which LoViReTs displayed a significant increase in quasispecies diversity (LoViReTs=1.2x10–^2^ vs. controls=4.6x10^-3^; *p*-adj. = 0.05), contrasting with the pattern observed for *env* and *gag*. These unexpected differences between genomic regions prompted further investigation into the factors underlying the elevated *pol* variability in LoViReTs to clarify the distinct behavior of this genomic segment.

### APOBEC-driven mutations were frequent in pol gene regions post-ART

Region-specific analyses reported no significant apolipoprotein B mRNA editing enzyme catalytic polypeptide-like (APOBEC)-mediated hypermutation in the *gag* or *env* regions. However, analysis of *pol* quasispecies revealed an excess of G-to-A substitutions in sequences from a subset of LoViReT participants, suggestive of APOBEC-mediated hypermutation. Although this increased hypermutation was observed only in a proportion of LoViReTs, the overall frequency of APOBEC-associated mutations was significantly higher than that in controls (*p*-adj. = 0.03) (**Figure 2C**). This pattern may contribute to the greater genetic diversity found in the *pol* locus during ART (**Figures 1B**, **2B**).

### LoViReTs showed lower viral diversity and limited evolution even before treatment initiation

For participants with matched pre- and post-ART samples available (LoViReTs n=7; controls n=8), we repeated the analyses to assess whether the evolutionary dynamics observed in LoViReTs during ART were already evident prior to treatment initiation.

Overall Shannon entropy across all regions was significantly lower in LoViReTs during therapy within the matched-samples subset (*p*-adj. = 0.0007; **Figure 3A**), consistent with observations in the full cohort (**Figure 1A**). Pre-ART samples showed a similar, although non-significant, trend toward reduced entropy in LoViReTs compared with controls (*p*-adj. = 0.11; **Figure 3A**). Gene-specific assessment also indicated a similar tendency toward lower entropy in *env* (*p*-adj. = 0.15; **Figure 3B**), despite the limited dataset. These patterns were mirrored when analyzing weighted quasispecies diversity via average gene diversity between pre- and post-ART matched samples, with larger differences observed in the *env* region, although not statistically significant (**Figure 3C**).

**Figure 3:**
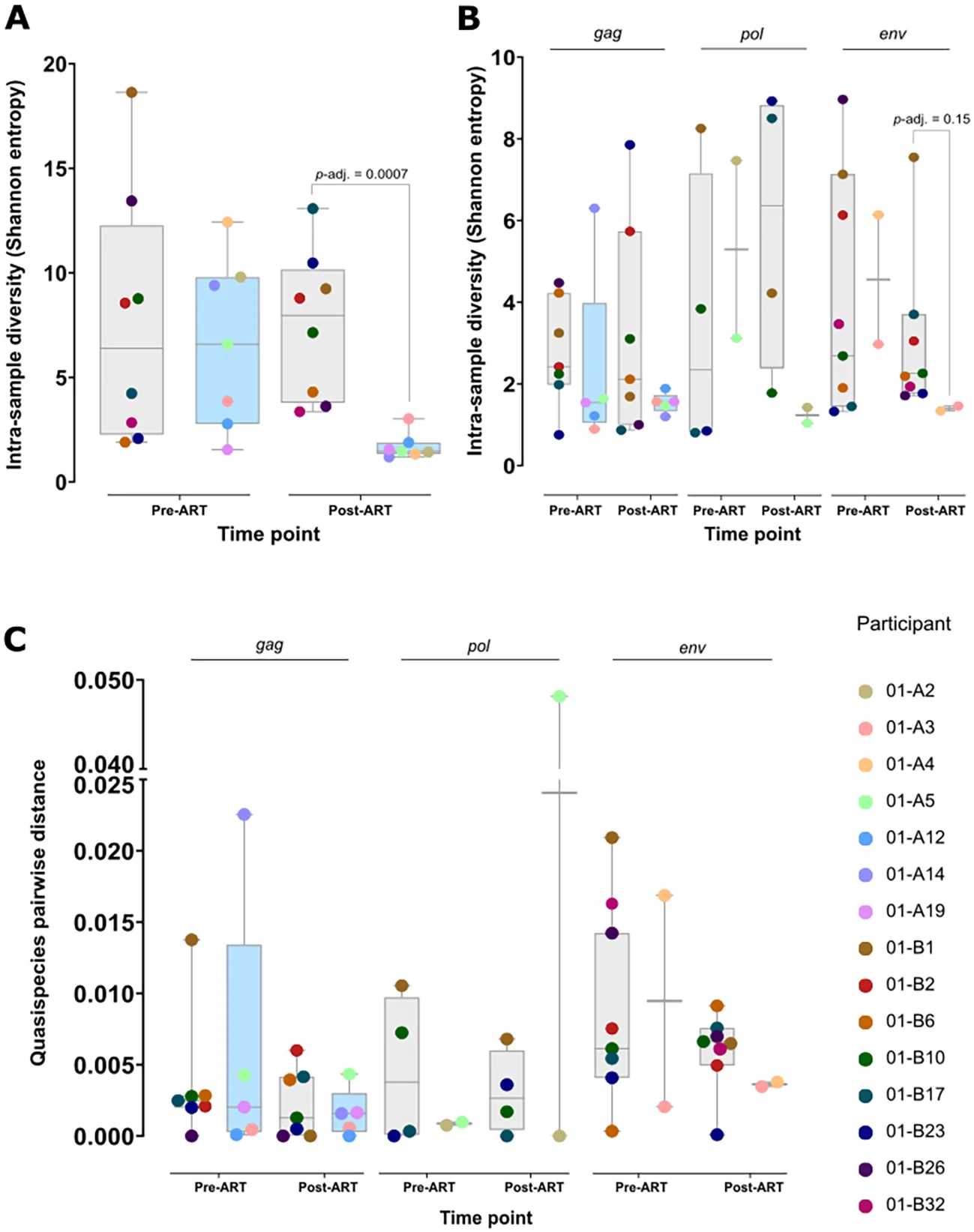
Intra-sample diversity and quasispecies pairwise distance before and after ART. **(A)** Shannon entropy values per time point (pre-ART versus post-ART). **(B)** Shannon entropy by gene region shows that LoViReTs maintain lower diversity on ART, primarily driven by the *env* gene. **(C)** Gene-specific diversity. LoViReTs generally maintain lower quasispecies pairwise distances than controls at both time points. LoViReTs (<100 HIV-1-DNA copies/10^6^ CD4^+^ T-cells) are highlighted in blue, while controls (>100 HIV-1-DNA copies/10^6^ CD4^+^ T-cells) are colored in light gray. Pre-ART and post-ART matched samples are color-coded per participant.

Furthermore, time-normalized root-to-tip distances within *env*, calculated using the 2021 Consensus B sequence as reference, showed that *env* consensus sequences from controls accumulated additional evolutionary divergence during ART, whereas no such increase was detected in LoViReTs (*p* = 0.09; **Supplementary Figure 3**). Although limited by the small number of LoViReTs included in the analysis, this pattern suggested a disrupted evolutionary capacity in LoViReTs.

### Associations between HIV-1 reservoir genetic characteristics and clinical variables

To examine factors associated with the restricted viral diversity observed in LoViReTs, independently of group classification, we explored relationships between their reservoir genetic characteristics during ART and several clinical features previously studied by our group in LoViReT participants (i.e., higher nadir CD4^+^ and early ART initiation, particularly with an InSTI-containing regimen) (10). Variables present in >90% of participants were considered, and the most parsimonious subset was selected by stepwise selection to fit a multivariable regression model that best explained reservoir diversity across the dataset. Total viral entropy levels were significantly associated with age at diagnosis (*p* = 0.032) and route of transmission (RoT) (*p* = 0.04; **Table 2A**). Stratified analysis by RoT further revealed that LoViReTs maintained relatively stable entropy values regardless of transmission modes, whereas controls, particularly people who inject drugs (PWID), displayed a broader distribution and higher entropy levels. Although limited by the small number of participants within some RoT categories, these findings pointed to differences in patterns of viral evolution according to transmission context among non-LoViReT individuals (**Figure 4A**), the relatively stable entropy observed in LoViReTs across transmission modes was consistent with the presence of highly efficient viral control mechanisms that may constrain viral evolution, potentially involving genetic or immune-related factors. Despite the inclusion of protective HLA alleles in the final model, their effect did not reach statistical significance. In contrast, applying the same approach to examine factors associated with *pol* hypermutation rates revealed a significant association between protective HLA alleles and APOBEC-mediated hypermutants (*p* = 0.007; **Table 2B**).

**Table 2:** Multivariable regression model.

A. Multivariate model with variables present in >90% of samples			
Characteristic	Beta	95% CI	*p*-value
Age at diagnosis	1.74x10^-1^	1.63x10^-2^, 3.32x10^-1^	0.032*
timeDtoART	-1.52x10^-1^	-3.23x10^-1^, 1.85x10^-2^	0.078
minCD4	-1.23x10^-1^	-2.88x10^-1^, 4.06x10^-2^	0.13
score_6protectiveHLAs	-1.17x10^-1^	-2.76x10^-1^, 4.20x10^-2^	0.14
Route of transmission			0.04*
MSM	–	–	
HET	1.12x10^-1^	-2.38x10^-1^, 4.62x10^-1^	
PWID	6.08x10^-1^	1.41x10^-1^, 1.08x10^0^	

B. *pol* gene hypermutation multivariate model			
Characteristic	Beta	95% CI	*p*-value
timeDtoS	1.73x10^-2^	-5.56x10^-6^, 3.46x10^-2^	0.05
score_6protectiveHLAs	3.31x10^-1^	9.78x10^-2^, 5.65x10^-1^	0.007*
PI	-8.68x10^-5^	-1.82x10^-4^, 8.69x10^-6^	0.073
InSTI	-1.60x10^-4^	-3.91x10^-4^, 7.15x10^-5^	0.2

Multivariate regression analysis of clinical and genetic factors associated with (A) viral diversity and (B) *pol* APOBEC-mediated hypermutation rates in LoViReTs during ART.

Abbreviations: CI, confidence interval; HET, heterosexual; HLA, human leukocyte antigen; InSTI, integrase strand transfer inhibitor; minCD4, minimum CD4^+^ T-cell counts (or nadir); MSM, men who have sex with men; PI, protease inhibitor; PWID, people who inject drugs; timeDtoART, time from HIV-1 diagnosis to initiation of ART; timeDtoS, time from HIV-1 diagnosis to sampling. *Statistically significant difference of *p*<0.05.

**Figure 4:**
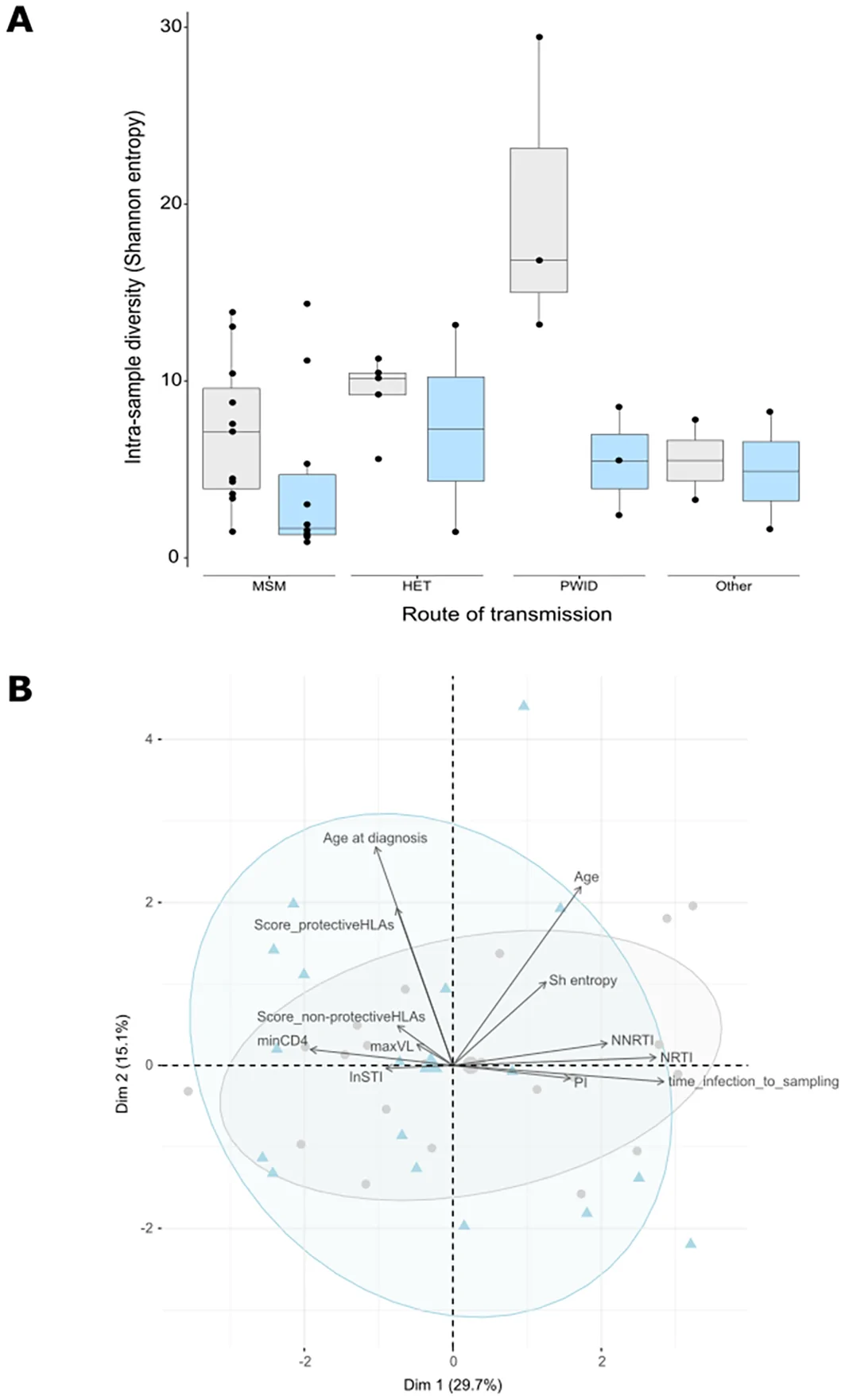
Multivariate analysis of HIV-1 diversity and clinical variables. **(A)** Shannon entropy across routes of transmission, with LoViReTs showing stable diversity and controls—especially PWID—exhibiting higher values. **(B)** Principal component analysis (PCA) of post-ART variables, highlighting LoViReT and control clustering. Variables include clinical parameters, proviral metrics, ART regimen, and HLA protective and non-protective scores. LoViReTs (<100 HIV-1-DNA copies/10^6^ CD4^+^ T-cells) are highlighted in blue, while controls (>100 HIV-1-DNA copies/10^6^ CD4^+^ T-cells) are colored in light gray. HET, heterosexual; MSM, men who have sex with men; PWID, people who inject drugs.

As a complementary approach, we performed a principal component analysis (PCA) to identify the variables that best explained the observed variability in our dataset, independently of group assignment. Using post-ART clinical and genetic data available for all participants, the PCA identified variables contributing to the observed variability in the dataset, although substantial overlap between groups remained evident (**Figure 4B**). LoViReTs clustered primarily based on protective HLA alleles and clinical metrics such as nadir CD4^+^ count, ART regimen, and age, consistent with previous observations (10). Protective allele frequencies were similar between groups (32% LoViReTs vs. 29% controls; *p* = 0.9), whereas risk alleles appeared significantly enriched in controls (52% vs. 26% LoViReTs; *p* = 0.01). Controls were further characterized by increased viral entropy and longer infection duration.

### HLA-restricted gag epitopes evolution related to the HIV-1 reservoir

Given the potential link between participants’ HLA profiles and the restricted viral diversity observed in LoViReTs, we conducted a focused analysis of HLA-associated epitope motifs within the *gag* region to assess evidence of viral escape or HLA-driven selection, reflected by enrichment of susceptible variants. This analysis was restricted to 8 participants (5 LoViReTs and 3 controls) for whom paired pre- and post-ART sequences were available, allowing longitudinal comparisons. Using the HIV LANL Motif Scan tool (21), we matched each participant’s HLA alleles to their corresponding *gag*-region peptide motifs in paired samples and examined changes in motif frequencies over time to identify HLA-associated epitope motif sites with frequency shifts and compare patterns across groups.

A complete list of all detected motifs and their frequency changes is provided in **Supplementary Table 3**. Across participants, variations in HLA motifs between pre- and post-ART samples were primarily driven by shifts in relative motif frequencies within the viral population, rather than by wholesale loss of pre-existing motifs or the emergence of novel ones. Although differences in most motifs did not reach statistical significance after correction for multiple testing, their distribution revealed heterogeneous patterns across individuals and groups after ART, offering exploratory insights into individual-level variation. Controls displayed widespread reductions in motif frequencies, whereas LoViReTs exhibited enrichment in selected motifs, reflecting intrinsically distinct evolutionary trajectories.

Despite the limited sample size, three epitopes from this broader set exhibited robust, highly significant changes and were therefore prioritized for further characterization (**Table 3**). Among them, one motif was enriched in LoViReTs, while two were more prominent in controls. Specifically, two of three controls revealed significant reductions in the frequency of motifs linked to HLA-B*35:01 and HLA-Cw*04:01 (x-PAV-x-x-x-x-x-x-x and x-YP-x-x-x-x-x-x-x, respectively). These decreases reflected amino-acid substitutions between pre- and post-ART samples at individual Gag positions overlapping HLA-restricted epitopes, predicted to disrupt antigen presentation and supporting ongoing viral adaptation with the capacity to accumulate immune-driven escape mutations. By contrast, LoViReTs showed no comparable reductions; instead, most exhibited significant enrichment of the HLA-A*02:01-associated motif (x-LM-x-x-x-x-x-x-VL), often present within sequences containing stop codons, suggesting limited potential for productive translation. Three of four LoViReTs with this motif displayed clear gains after ART, while one remained stable, indicating persistence and enrichment rather than escape (**Table 3**).

**Table 3:** Significant HLA motifs.

Motif	HLA genotype	Observations (n)	Position(s) with significant changes	Estimate	*p*-value	Adj. *p*-value	Participants (n)	Participant ID	Pre-ART Mean Freq.	Post-ART Mean Freq.	Freq. Change	Individual contribution to overall motif trend
x-LM-x-x-x-x-x-x-VL	A*02:01	114	10-18	-0,96	5,70E-10	1,71E-09	4	A3	0,5000	0,5000	0,0000	Freq. UNCHANGES
								A5	0,0646	0,2500	0,1854	Freq. INCREASES on ART (pre-ART < post-ART)
								A12	0,5000	1,0000	0,5000	Freq. INCREASES on ART (pre-ART < post-ART)
								A14	0,0667	0,5000	0,4333	Freq. INCREASES on ART (pre-ART < post-ART)
x-PAV-x-x-x-x-x-x-x	B*35:01	1276	16-24; 91-99; 155-163	0,612	1,30E-28	1,17E-27	2	B10	0,0976	0,0186	-0,0790	Freq. DECREASES on ART (pre-ART > post-ART)
								B17	0,2500	0,2484	-0,0016	Freq. DECREASES on ART (pre-ART > post-ART)
x-YP-x-x-x-x-x-x-x	Cw*04:01	414	78-86	0,627	1,27E-10	5,73E-10	2	B10	0,0976	0,0186	-0,0790	Freq. DECREASES on ART (pre-ART > post-ART)
								B17	0,2500	0,2484	-0,0016	Freq. DECREASES on ART (pre-ART > post-ART)

List of HLA-associated motifs showing statistically significant frequency variations (*p* < 0.05) during ART.

A, LoViReT; ART, antiretroviral therapy; adj., FDR-adjusted; B, Control; FC, fold change; HLA, human leukocyte antigen.

Overall, these findings reinforced the notion that controls retained measurable evolutionary flexibility within their proviral reservoirs under ART, whereas LoViReTs reflected markedly constrained viral dynamics and limited ability to adapt to or evade host immune pressure.

## Discussion

While HIV-1 reservoir quantification and persistence have been extensively studied in ART-treated individuals, the extent and functional relevance of genetic diversity, including its qualitative genomic features, remain insufficiently characterized, particularly in PWH with exceptionally low reservoirs. Assessment of viral diversity provides valuable insights into the mechanisms underlying reservoir establishment and maintenance, which could inform strategies to reduce reservoir size. Notably, immunotherapeutic interventions may be more effective against genetically homogeneous reservoirs compared with highly diverse ones (36, 37).

We have previously described and explored the relationship between various clinical factors and reservoir size in LoViReTs—a unique, well-defined group of PWH on ART who maintain extremely low HIV-1-DNA levels, even prior to treatment initiation (9). These individuals exhibited altered reservoir distribution among short-lived CD4^+^ T-cell subsets (8), and their reservoir levels have been correlated with earlier ART initiation, particularly with InSTIs, as well as higher nadir CD4^+^ counts (10). However, the factors identified to date do not fully account for the LoViReT phenotype, highlighting the need for further virological and immunological investigation to elucidate the mechanisms of restricted latency in this group.

In this study, we selected a subgroup of LoViReTs to examine the association between HIV-1 genetic diversity and reservoir characteristics. We compared viral sequences between LoViReT and control participants before and after long-term ART, analyzing viral genetic features across both time points.

Nucleotide-level sequence diversity, measured by Shannon entropy, revealed that LoViReTs possess significantly less genetically diverse intra-host reservoirs, supporting the association between greater HIV-1 population diversity and larger reservoir size (7, 37, 38). Viral diversity appears to facilitate persistence and adaptation to immune responses, ART, and host environments, thereby complicating efforts to eradicate HIV-1 (39). Studies in ART-naïve individuals have shown substantial entropy heterogeneity across viral proteins, with Env displaying the highest sequence diversity, whereas Pol remains relatively conserved (40). Our findings are consistent with these observations, as the most pronounced differences were detected in the *env* gene, particularly after prolonged ART.

Reference-based quasispecies mapping further demonstrated this altered diversification, with LoViReTs displaying highly uniform reservoirs dominated by a single haplotype. This aligns with evidence that pre-ART homogeneous, low-diversity viruses tend to establish smaller, more stable reservoirs that are less prone to viral rebound, resulting in improved long-term treatment outcomes (41–43). Our phylogenetic and diversity analyses reinforce these findings, reporting reduced viral diversity in LoViReTs, especially during ART, which further restricts viral expansion. This was particularly evident in the *gag* and *env* genes, which displayed minimal diversity under treatment.

Unexpectedly, the *pol* gene exhibited greater diversity—especially during ART—despite expectations that *env* would show the highest variability due to its role in viral entry, the strong immune pressure exerted by neutralizing antibodies, and its higher mutation rates in gp41 (44). *Pol* is generally more conserved than *gag* and *env* (45, 46). Thus, we first discarded the possibility that increased *pol* diversity resulted from primers targeting drug resistant-associated regions (47, 48) (data not shown). Instead, we found that LoViReTs exhibited higher frequencies of APOBEC-mediated hypermutation compared with controls. This observation is consistent with previous reports indicating increased HIV-1 mutation rates in individuals with typical disease progression compared with rapid progressors, suggesting that APOBEC3G-driven hypermutation negatively affects viral fitness (44). Accordingly, APOBEC-mediated hypermutation is likely dominated by APOBEC3G—the principal and most potent host cytidine deaminase responsible for G-to-A hypermutation in HIV-1 (49)—although contributions from other APOBEC enzymes cannot be excluded. In LoViReTs, APOBEC-induced hypermutation was more pronounced in *pol* than in *gag* or *env*. Prior studies have documented variable hypermutation across the genome, with modest G-to-A substitutions in *gag*, increasing toward the *pol* RNAse domain, dropping at the 3’ end of integrase, and rising again near the end of *env* gp41 before declining (50). Our results align with this pattern, as the *env* sequencing primers targeted a 300 bp segment of the gp120 ectodomain not enriched for APOBEC3G sites (51), likely explaining the lack of APOBEC impact on LoViReTs’ HIV-1 evolution in this gene. Comparison with prior studies (52, 53) suggests that the amplified *pol* region falls within a hypermutation hotspot of the HIV-1 genome, which may contribute to the observed sequence variability. Future research is warranted to clarify the role of APOBEC in shaping the restricted reservoir of LoViReTs.

Multivariate analysis integrating clinical, virological, and host genetic data revealed distinct profiles for LoViReTs and controls. LoViReTs clustered with protective HLA alleles, early diagnosis, and reduced viral diversity, whereas controls—particularly PWID —had higher viral entropy, longer infection duration, and larger proviral burdens. The increased diversity observed in PWID is consistent with previous studies demonstrating that greater multiplicity of infection and exposure to more heterogeneous quasispecies leads to more complex reservoirs compared with sexually transmitted cases (54), even under ART. Interestingly, the inverse relationship between the number of protective HLA alleles and viral entropy supports the hypothesis that HLA-mediated immune pressure constrains viral diversity. This association is well-established, as HLA-B*57 and HLA-B*27 alleles have been associated with effective, durable HIV-1 control through robust cytotoxic T lymphocyte (CTL) responses that limit viral evolution and disease progression by reducing viral escape and replication without compromising viral fitness (55). Our exploratory analysis of HLA-associated motifs extended this concept by suggesting allele-specific dynamics in viral evolution, although protective alleles were not clearly overrepresented in our study group, and these observations warrant validation in larger cohorts. In controls, motifs linked to detrimental HLA alleles, including HLA-B*35:01 and HLA-Cw*04:01, showed significant reductions in frequency, consistent with viral adaptation under immune pressure. These alleles have been linked to poor immune control, higher viral loads, and rapid selection of escape mutations, reflecting ongoing viral evolution (56, 57). In contrast, LoViReTs showed enrichment of motifs associated with HLA-A*02:01, an allele with complex, context-dependent effects. While it can promote strong, often polyfunctional HIV-1-specific CD8^+^ T-cell responses in certain settings (58, 59) and has been associated with uninfected controls (60), other studies link it to higher viral loads and low CD4^+^ T-cell counts (57). This suggests that the role of HLA-A*02:01 in long-term control is uncertain and unlikely to independently drive the viral suppression seen with other protective alleles. Notably, prior work from our group in LoViReT participants demonstrated minimal inhibition of HIV-1 infection in CD4^+^ T-cells by autologous CD8^+^ or NK cells (9), suggesting that the restricted reservoir may arise from the combined influence of host genetics and limited functional antiviral immune responses. Collectively, these findings underscore the critical interplay between protective HLA genotypes and immune pressure in preventing viral diversification. This limited evolution ultimately results in the small, genetically restricted, and functionally impaired viral reservoir characteristic of LoViReTs, providing little to no evolutionary potential for adaptive immune escape following treatment. Yet, further research is needed to clarify the relative contributions of host genetics and cellular immunity in shaping the LoViReT phenotype.

A key limitation of this study is the small reservoir size in LoViReTs, which constrained HIV-1 amplification and sequencing and may have limited the detection of low-frequency variants, potentially leading to an underestimation of viral diversity. To mitigate this, we used multiple primer sets targeting different regions of *gag*, *pol*, and *env*, maximizing amplification despite not capturing complete gene sequences. However, these selected subgenomic regions may be subject to region-specific differences in sequence diversity and APOBEC-associated hypermutation hot spots, as observed in the *pol* region. Therefore, our findings should be interpreted as evidence of restricted diversity within the regions analyzed rather than as a genome-wide assessment of HIV-1 diversity. Importantly, the short-amplicon approach does not allow us to determine proviral intactness or distinguish intact from defective proviruses, which may be subject to distinct evolutionary and immune-selective pressures. Thus, the observed differences in viral diversification should be interpreted as characteristics of the amplified proviral population and, although consistent with differential immune-mediated selection, cannot be specifically attributed to intact or replication-competent proviruses. Moreover, although ART initiation timing was comparable between groups, the potential influence of early versus chronic ART on viral diversification cannot be completely excluded and should be considered when interpreting our findings. Finally, the HLA-associated motif analysis was exploratory and limited by the small number of participants available for these comparisons; therefore, these findings should be interpreted cautiously and require validation in larger cohorts.

In summary, LoViReTs harbor significantly lower viral diversity compared with controls despite similar pre-ART viral loads and treatment regimens. This reduced diversity was associated with restricted reservoirs and higher levels of APOBEC-associated hypermutation, particularly in *pol*, and may be consistent with stronger host-mediated immune pressure, potentially involving IFN-stimulated genes such as APOBEC3G. Our findings indicate that the viral populations detected in LoViReTs may have undergone more restricted evolutionary diversification, potentially limiting their capacity for adaptation. Additional studies using full-genome sequencing are needed to elucidate the immunovirological mechanisms underlying the LoViReT phenotype and determine the generalizability of these findings across the viral genome. These insights will inform strategies aimed at HIV-1 eradication and therapies designed to reduce the pool of latently infected cells, thereby advancing efforts toward a functional cure.

## Data Availability

The data presented in this study are deposited in the NIH repository under accession numbers 64328078-64328132.

## Supplementary material

Supplementary Figure 1 Primer location. Schematic representation of the HIV-1 genome, highlighting the primer binding sites used in a nested PCR approach to amplify specific sub-genomic regions of *gag* (blue), *pol* (green), and *env* (red) genes. Primer names are labeled beneath each amplified segment, with arrows indicating their orientation and position along the viral genome.

Supplementary Figure 2 HIV-1 quasispecies phylogenies. Maximum-likelihood tree of **(A)** *gag* and **(B)** *pol* quasispecies. Tips represent unique haplotypes, sized by frequency, and color-coded by participant and group. LoViReTs (<100 HIV-1-DNA copies/10^6^ CD4^+^ T-cells) are highlighted in blue, while controls (>100 HIV-1-DNA copies/10^6^ CD4^+^ T-cells) are colored in light gray. Hypermutants are indicated by line-dotted boxes.

Supplementary Figure 3 Evolutionary divergence. Time-normalized root-to-tip distances between amplified HIV-1 *gag*, *pol*, and *env* sequences and the 2021 Consensus B reference sequence, comparing LoViReT participants and controls.
