# Supplementary material for: Restricted HIV-1 diversity in people with HIV and exceptionally low viral reservoirs
Source: Front Immunol. 2026 Oct 30;17:1904455. doi: 10.3389/fimmu.2026.1904455 (PMC13759741; doi:10.3389/fimmu.2026.1904455)

**Supplementary Table 1. Gene amplification.** Summary of viral genes successfully amplified from pre- and post-ART samples for each study participant. 01 and 02 indicate follow-up at Hospital Germans Trias i Pujol and Hospital Clinic, respectively.

| Participant ID | Tropism | Subtype | Max. VL | Gene amplified pre-ART | | | Gene amplified post-ART | | |
|---|---|---|---|---|---|---|---|---|---|
| | | | | *gag* | *pol* | *env* | *gag* | *pol* | *env* |
| 01-A1 | | F1 | 39428 | | x | | | | |
| 01-A2 | CCR5 | B | 68000 | x | x | x | | x | |
| 01-A3 | CCR5 | B | 180000 | x | x | x | x | | x |
| 01-A4 | | | 59000 | x | | x | | | x |
| 01-A5 | | | 120000 | x | x | x | x | x | |
| 01-A8 | CCR5 | B | 244 | | | | x | x | x |
| 01-A10 | CCR5 | B | 14000 | | | | | x | |
| 01-A12 | CCR5 | B | 600 | x | x | x | x | | |
| 01-A13 | CCR5 | B | 3200 | | | | x | | |
| 01-A14 | CCR5 | B | 85000 | x | | x | x | | |
| 01-A15 | | | 401000 | x | x | x | | | |
| 01-A17 | | | 8100 | | | | x | x | x |
| 01-A19 | CCR5 | B | 4300 | x | | | x | | |
| 01-A22 | CCR5 | C | 37000 | | | | x | x | x |
| 02-A1 | | | 5978 | | | | | x | x |
| 02-A2 | CXCR4 | B | 3557 | | | | x | x | x |
| 02-A3 | CCR5 | B | 89 | | | | | x | x |
| 02-A5 | | | 32900 | | | | x | x | |
| 02-A6 | CCR5 | B | 50620 | | | | x | x | x |
| 01-B1 | CCR5 | B | 181000 | x | x | x | x | x | x |
| 01-B2 | CCR5 | B | 120000 | x | | x | x | | x |
| 01-B3 | CCR5 | B | 47500 | | | | x | x | x |
| 01-B4 | CCR5 | A/AG | 73263 | | | | x | x | x |
| 01-B5 | CXCR4 | B | 50 | | | | x | x | x |
| 01-B6 | CCR5 | B | 840 | x | x | x | x | | x |
| 01-B7 | CCR5 | B | 5300 | | | | x | x | x |
| 01-B8 | CCR5 | B | 9600 | | | | x | x | x |
| 01-B10 | CCR5 | B | 32000 | x | x | x | x | x | x |
| 01-B12 | CCR5 | B | 23000 | | | | x | x | x |
| 01-B13 | CCR5 | B | 400 | | | | x | x | x |
| 01-B17 | CCR5 | B | 42000 | x | x | x | x | x | x |
| 01-B18 | CCR5 | B | 1780 | | | | x | x | x |
| 01-B20 | CCR5 | B | 52000 | | | | x | x | x |
| 01-B21 | CCR5 | B | 170000 | | | | x | x | x |
| 01-B23 | CXCR4 | B | 9100 | x | x | x | x | x | x |
| 01-B25 | CCR5 | B | 199000 | | | | x | x | x |
| 01-B26 | CCR5 | B | 12000 | x | | x | x | | x |
| 01-B28 | | | 120000 | | | | | x | |
| 01-B30 | CXCR4 | B | 750000 | | | | x | x | x |
| 01-B32 | CCR5 | B | 190000 | | | x | | | x |

Abbreviations: A, LoViReT; ART, antiretroviral therapy, B, control; VL, viral load (in HIV-1-RNA copies per mL)

**Supplementary Table 2. Nested-PCR primer characteristics.** List of primers used for amplification of *gag*, *pol*, and *env* regions, including amplicon size, genomic position, and sequence.

| Target gene | Nested PCR | Amplicon size (bp) | Primer | Primer name | Amplified region (bp) | Primer length (bp) | Primer sequence (5' → 3') |
|---|---|---|---|---|---|---|---|
| Group specific antigen (*gag*) | Outer | 1117 | Forward | *gag*_1 | 632 - 647 | 16 | GCAGTGGCGCCCGAAC |
| | | | Reverse | *gag*_4 | 1765 - 1788 | 24 | AATAGTCTTACAATCTGGGTTCGC- |
| | Inner | 540 | Forward | *gag*_2 | 978 - 1000 | 23 | GCTACAACCAKCCCTYCAGACAG |
| | | | Reverse | *gag*_3 | 1541 - 1564 | 24 | CTACTGGGATAGGTGGATTAYKTG |
| Polymerase (*pol*) | Outer | 1350 | Forward | *pol*_1 | 2137 - 2156 | 20 | CAGAGCAGACCAGAGCCAAC |
| | | | Reverse | *pol*_4 | 3507 - 3531 | 25 | CTGCTATTAAGTCTTTTGATGGGTC |
| | Inner | 465 | Forward | *pol*_2 | 2235 - 2257 | 23 | ACTCTATCCTTTAGCTTCCCTCA |
| | | | Reverse | *pol*_3 | 2723 - 2744 | 22 | TTTCTTTATGGCAAATACTGGA |
| Envelope (*env*) | Outer | 538 | Forward | *env*_1 | 6953 - 6974 | 22 | CACAGTACAATGTACACATGGA |
| | | | Reverse | *env*_4 | 7513 - 7535 | 23 | AGGGGCATACATTGCTTTTCCTA |
| | Inner | 283 | Forward | *env*_2 | 7009 - 7030 | 22 | ATGGCAGTCTAGCAGAAGAAGA |
| | | | Reverse | *env*_3 | 7314 - 7334 | 21 | TTCTGGGTCCCCTCCTGAGGA |

Amplicon length and alignment information was extracted from QuickAlign online open tool from Los Alamos National Laboratory Sequence Database available at https://www.hiv.lanl.gov/ (accessed 03.03.2025).

**Supplementary Table 3. Participant-specific HLA-associated motifs.** Summary of frequency changes in HLA-associated motifs across participants. Motif-associated HLA alleles in bold indicate statistically significant frequency variations at those sites.

| Participant ID | Total changing positions | ↑ Frequency | ↓ Frequency | New | Lost | Unchanged | Full HLA genotype | Motif-associatied HLA alleles | Motif | Positions (n) | Pre-ART Mean Freq. | Post-ART Mean Freq. | Freq. Change | Direction of change |
|---|---|---|---|---|---|---|---|---|---|---|---|---|---|---|
| A3 | 41 | 0 | 0 | 0 | 0 | 41 | A*02:01/24:02<br>B*18:01/51:01<br>C*02:02/07:01<br>DRB1*11:04/15:01<br>DQB1*06:01/06:02 | **A*02:01** | **x-LM-x-x-x-x-x-x-VL** | 3 | 0.5000 | 0.5000 | 0.0000 | Stable |
| | | | | | | | | B*51:01 | x-APGWF-x-x-x-x-x-x-VIWMVL | 7 | 0.5000 | 0.5000 | 0.0000 | Stable |
| | | | | | | | | | x-x-x-x-x-x-x-x-FI | 11 | 0.5000 | 0.5000 | 0.0000 | Stable |
| | | | | | | | | DRB1*15:01 | L-V-I-x-x-FYI-x-x-ILVMF | 1 | 0.5000 | 0.5000 | 0.0000 | Stable |
| | | | | | | | | DQB1*06:02 | AFCILMNQSTVWYDE-x-AFGILMNQSTVWYCDE-AFGILMNQSTVWY-x-LIVAPST-x-x-ASTGLIVP | 19 | 0.5000 | 0.5000 | 0.0000 | Stable |
| A5 | 35 | 35 | 0 | 0 | 0 | 0 | A*02:01/11:01<br>B*40:01/45:01<br>C*03:04/16:01<br>DRB1*01:02/10:01<br>DQB1*05:01/05:01 | **A*02:01** | **x-LM-x-x-x-x-x-x-VL** | 3 | 0.0646 | 0.2500 | 0.1854 | Increased |
| | | | | | | | | A*11:01 | x-x-x-x-x-x-x-x-K | 11 | 0.0646 | 0.2500 | 0.1854 | Increased |
| | | | | | | | | | x-x-x-x-x-x-x-x-KR | 17 | 0.0651 | 0.2640 | 0.1989 | Increased |
| | | | | | | | | B*40:01 | x-E-x-x-x-x-x-x-L | 2 | 0.0646 | 0.2500 | 0.1854 | Increased |
| | | | | | | | | B*45:01 | ILVM-x-x-ALM-x-AGSTCP-x-x-ILAMYW | 1 | 0.0646 | 0.2500 | 0.1854 | Increased |
| | | | | | | | | | ILVM-x-x-ALM-x-AGSTP-x-x-ILAMYW | 1 | 0.0646 | 0.2500 | 0.1854 | Increased |
| A12 | 22 | 16 | 0 | 6 | 0 | 0 | A*02:01/03:01<br>B*15:01/15:01<br>C*02:02/03:03<br>DRB1*04:01/11:01<br>DQB1*03:01/03:02 | **A*02:01** | **x-LM-x-x-x-x-x-x-VL** | 5 | 0.5000 | 1.0000 | 0.5000 | Increased |
| | | | | | | | | | | 5 | 0.0000 | 1.0000 | 1.0000 | New event |
| | | | | | | | | A*03:01 | x-LVMIAST-x-x-x-x-x-x-KYFR | 5 | 0.5000 | 1.0000 | 0.5000 | Increased |
| | | | | | | | | B*15:01 | FLV-x-x-x-x-x-x-x-NQST | 6 | 0.5000 | 1.0000 | 0.5000 | Increased |
| | | | | | | | | | | 1 | 0.0000 | 1.0000 | 1.0000 | New event |
| A14 | 73 | 13 | 0 | 31 | 29 | 0 | A*02:01/68:01<br>B*35:03/49:01<br>C*04:01/07:01<br>DRB1*01:01/04:07<br>DQB1*03:01/05:01 | **A*02:01** | **x-LM-x-x-x-x-x-x-VL** | 2 | 0.0667 | 0.5000 | 0.4333 | Increased |
| | | | | | | | | | | 4 | 0.0000 | 0.5000 | 0.5000 | New event |
| | | | | | | | | | | 2 | 0.0667 | 0.0000 | -0.0667 | Lost event |
| | | | | | | | | A*68:01 | x-VT-x-x-x-x-x-x-RK | 2 | 0.0667 | 0.5000 | 0.4333 | Increased |
| | | | | | | | | | | 2 | 0.0000 | 0.5000 | 0.5000 | New event |
| | | | | | | | | B*35:03 | x-PA-x-x-x-x-x-x-MLF | 1 | 0.0000 | 0.5000 | 0.5000 | New event |
| | | | | | | | | | | 1 | 0.0667 | 0.0000 | -0.0667 | Lost event |
| | | | | | | | | | x-PMILFV-x-x-x-x-x-x-MLF | 3 | 0.0667 | 0.5000 | 0.4333 | Increased |
| | | | | | | | | | | 5 | 0.0000 | 0.5000 | 0.5000 | New event |
| | | | | | | | | | | 6 | 0.0667 | 0.0000 | -0.0667 | Lost event |
| | | | | | | | | Cw*04:01 | x-YPF-x-x-x-x-x-x-LFM | 2 | 0.0000 | 0.5000 | 0.5000 | New event |

| | | | | | | | | | | | | | | |
|---|---|---|---|---|---|---|---|---|---|---|---|---|---|---|
| | | | | | | | | | | 2 | 0.0667 | 0.0000 | -0.0667 | Lost event |
| | | | | | | | | | x-YP-x-x-x-x-x-x-x | 4 | 0.0667 | 0.5000 | 0.4333 | Increased |
| | | | | | | | | | | 7 | 0.0000 | 0.5000 | 0.5000 | New event |
| | | | | | | | | | | 7 | 0.0667 | 0.0000 | -0.0667 | Lost event |
| | | | | | | | | DRB1*01:01 | YFWLIMVA-x-x-LMAIVN-x-AGSTCP-x-x-LAIVNFYMW | 5 | 0.0000 | 0.5000 | 0.5000 | New event |
| | | | | | | | | | | 6 | 0.0667 | 0.0000 | -0.0667 | Lost event |
| | | | | | | | | | YVLFIAMW-x-x-LAIVMNQ-x-AGSTCP-x-x-LAIVNFY | 5 | 0.0000 | 0.5000 | 0.5000 | New event |
| | | | | | | | | | | 5 | 0.0667 | 0.0000 | -0.0667 | Lost event |
| | | | | | | | | DRB1*04:07 | FYWxxAVKxNTDSxxxQN | 1 | 0.0667 | 0.5000 | 0.4333 | Increased |
| | | | | | | | | | | 1 | 0.0667 | 0.5000 | 0.4333 | Increased |
| A19 | 63 | 0 | 0 | 12 | 10 | 41 | A*01:01/03:01 B*49:01/57:01 C*07:01/07:02 DRB1*07:01/09:01 DQB1*03:03/03:03 | A*01:01 | x-x-DE-x-x-x-x-x-Y | 1 | 0.2500 | 0.2500 | 0.0000 | Stable |
| | | | | | | | | A*03:01 | x-LVMIAST-x-x-x-x-x-x-KYFR | 2 | 0.0000 | 0.2500 | 0.2500 | New event |
| | | | | | | | | | | 5 | 0.2500 | 0.2500 | 0.0000 | Stable |
| | | | | | | | | B*57:01 | x-ATS-x-x-x-x-x-x-FW | 1 | 0.0000 | 0.2500 | 0.2500 | New event |
| | | | | | | | | | | 2 | 0.2500 | 0.0000 | -0.2500 | Lost event |
| | | | | | | | | | | 1 | 0.2500 | 0.2500 | 0.0000 | Stable |
| | | | | | | | | | x-ATS-x-x-x-x-x-x-FWY | 1 | 0.0000 | 0.2500 | 0.2500 | New event |
| | | | | | | | | | | 2 | 0.2500 | 0.0000 | -0.2500 | Lost event |
| | | | | | | | | | | 2 | 0.2500 | 0.2500 | 0.0000 | Stable |
| | | | | | | | | Cw*07:01 | x-RHK-x-x-x-x-x-x-Y | 1 | 0.2500 | 0.2500 | 0.0000 | Stable |
| | | | | | | | | Cw*07:02 | x-x-x-x-x-x-x-x-YFL | 1 | 0.0000 | 0.2500 | 0.2500 | New event |
| | | | | | | | | | | 1 | 0.2500 | 0.0000 | -0.2500 | Lost event |
| | | | | | | | | | | 14 | 0.2500 | 0.2500 | 0.0000 | Stable |
| | | | | | | | | DRB1*07:01 | FILVY-x-x-x-x-NST | 2 | 0.0000 | 0.3552 | 0.3552 | New event |
| | | | | | | | | | | 2 | 0.3292 | 0.0000 | -0.3292 | Lost event |
| | | | | | | | | | | 11 | 0.2500 | 0.2500 | 0.0000 | Stable |
| | | | | | | | | | FYWILV-x-x-DEHKNQRSTY-x-NST-x-x-VILYF | 1 | 0.0000 | 0.3218 | 0.3218 | New event |
| | | | | | | | | DRB1*09:01 | WYFL-x-x-AVS | 2 | 0.0000 | 0.3872 | 0.3872 | New event |
| | | | | | | | | | | 2 | 0.3085 | 0.0000 | -0.3085 | Lost event |
| | | | | | | | | | | 5 | 0.2500 | 0.2500 | 0.0000 | Stable |
| | | | | | | | | | YFWL-x-x-AS | 2 | 0.0000 | 0.3872 | 0.3872 | New event |
| | | | | | | | | | | 1 | 0.3670 | 0.0000 | -0.3670 | Lost event |
| | | | | | | | | | | 1 | 0.2500 | 0.2500 | 0.0000 | Stable |
| B10 | 70 | 0 | 68 | 2 | 0 | 0 | A*02:01/02:01 B*35:01/49:01 C*04:01/07:01 DRB1*08:01/11:02 DQB1*03:01/04:02 | A*02:01 | x-LM-x-x-x-x-x-x-VL | 4 | 0.0976 | 0.0186 | -0.0790 | Decreased |
| | | | | | | | | **B*35:01** | x-PAVYRD-x-x-x-x-x-x-YFMLI | 1 | 0.0000 | 0.0147 | 0.0147 | New event |
| | | | | | | | | | | 6 | 0.0976 | 0.0186 | -0.0790 | Decreased |
| | | | | | | | | | **x-PAV-x-x-x-x-x-x-x** | 1 | 0.0000 | 0.0196 | 0.0196 | New event |
| | | | | | | | | | | 40 | 0.0976 | 0.0186 | -0.0790 | Decreased |
| | | | | | | | | **Cw*04:01** | x-YPF-x-x-x-x-x-x-LFM | 2 | 0.0976 | 0.0186 | -0.0790 | Decreased |
| | | | | | | | | | **x-YP-x-x-x-x-x-x-x** | 13 | 0.0976 | 0.0186 | -0.0790 | Decreased |
| | | | | | | | | DRB1*08:01 | FILVY-x-x-x-HKR | 3 | 0.0976 | 0.0186 | -0.0790 | Decreased |

| | | | | | | | | | | | | | |
|---|---|---|---|---|---|---|---|---|---|---|---|---|---|
| B17 | 127 | 2 | 118 | 7 | 0 | 0 | A*11:01/31:01<br>B*07:02/35:01<br>C*04:01/07:02<br>DRB1*01:01/13:01<br>DQB1*05:01/06:03 | A*11:01 | x-x-x-x-x-x-x-x-K | 1 | 0.0000 | 0.4268 | 0.4268 New event |
| | | | | | | | | | | 10 | 0.2500 | 0.2484 | -0.0016 Decreased |
| | | | | | | | | | x-x-x-x-x-x-x-x-KR | 1 | 0.3691 | 0.5166 | 0.1475 Increased |
| | | | | | | | | | | 1 | 0.0000 | 0.4268 | 0.4268 New event |
| | | | | | | | | | | 15 | 0.2500 | 0.2484 | -0.0016 Decreased |
| | | | | | | | | A*31:01 | x-x-x-x-x-x-x-x-R | 1 | 0.3691 | 0.5166 | 0.1475 Increased |
| | | | | | | | | | | 5 | 0.2500 | 0.2484 | -0.0016 Decreased |
| | | | | | | | | B*07:02 | x-PV-x-x-x-x-x-x-L | 2 | 0.2500 | 0.2484 | -0.0016 Decreased |
| | | | | | | | | | x-P-x-x-x-x-x-x-LF | 1 | 0.2500 | 0.2484 | -0.0016 Decreased |
| | | | | | | | | **B*35:01** | x-PAVYRD-x-x-x-x-x-x-YFMLI | 6 | 0.2500 | 0.2484 | -0.0016 Decreased |
| | | | | | | | | | **x-PAV-x-x-x-x-x-x-x** | 1 | 0.0000 | 0.4268 | 0.4268 New event |
| | | | | | | | | | | 38 | 0.2500 | 0.2437 | -0.0063 Decreased |
| | | | | | | | | **Cw*04:01** | x-YPF-x-x-x-x-x-x-LFM | 2 | 0.2500 | 0.2484 | -0.0016 Decreased |
| | | | | | | | | | **x-YP-x-x-x-x-x-x-x** | 13 | 0.2500 | 0.1592 | -0.0909 Decreased |
| | | | | | | | | Cw*07:02 | x-x-x-x-x-x-x-x-YFL | 15 | 0.2500 | 0.2484 | -0.0016 Decreased |
| | | | | | | | | DRB1*01:01 | YFWLIMVA-x-x-LMAIVN-x-AGSTCP-x-x-LAIVNFYMW | 2 | 0.0000 | 0.4268 | 0.4268 New event |
| | | | | | | | | | | 6 | 0.2500 | 0.2484 | -0.0016 Decreased |
| | | | | | | | | | YVLFIAMW-x-x-LAIVMNQ-x-AGSTCP-x-x-LAIVNFY | 2 | 0.0000 | 0.4268 | 0.4268 New event |
| | | | | | | | | | | 5 | 0.2500 | 0.2484 | -0.0016 Decreased |
| B26 | 43 | 0 | 0 | 0 | 0 | 43 | A*24:02/66:01<br>B*07:02/41:02<br>C*07:02/17:03<br>DRB1*13:03/15:01<br>DQB1*03:01/06:02 | A*66:01 | x-TVAPLIC-x-x-x-x-x-x-RK | 3 | 1.0000 | 1.0000 | 0.0000 Stable |
| | | | | | | | | B*07:02 | x-PV-x-x-x-x-x-x-L | 3 | 1.0000 | 1.0000 | 0.0000 Stable |
| | | | | | | | | | x-P-x-x-x-x-x-x-LF | 1 | 1.0000 | 1.0000 | 0.0000 Stable |
| | | | | | | | | Cw*07:02 | x-x-x-x-x-x-x-x-YFL | 15 | 1.0000 | 1.0000 | 0.0000 Stable |
| | | | | | | | | DRB1*15:01 | LVI-x-x-FYI-x-x-ILVMF | 1 | 1.0000 | 1.0000 | 0.0000 Stable |
| | | | | | | | | DQB1*06:02 | AFCILMNQSTVWYDE-x-AFGILMNQSTVWYCDE-AFGILMNQSTVWY-x-LIVAPST-x-x-ASTGLIVP | 20 | 1.0000 | 1.0000 | 0.0000 Stable |

Abbreviations: A, LoViReT; ART, antiretroviral therapy, B, control; freq., frequency; HLA, human leukocyte antigen; LoViReT, Low Viral Reservoir Treated

## Supplementary Figure 1. Primer location

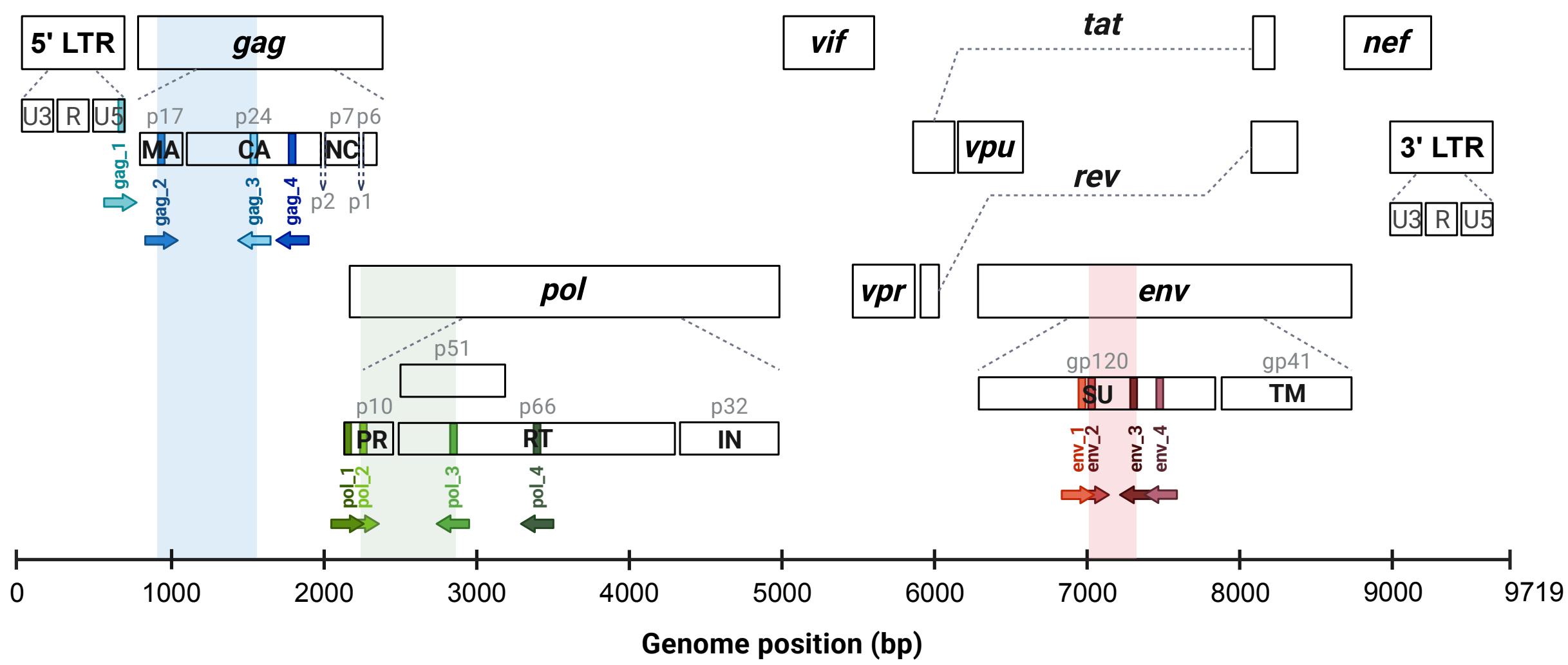

# Supplementary Figure 2

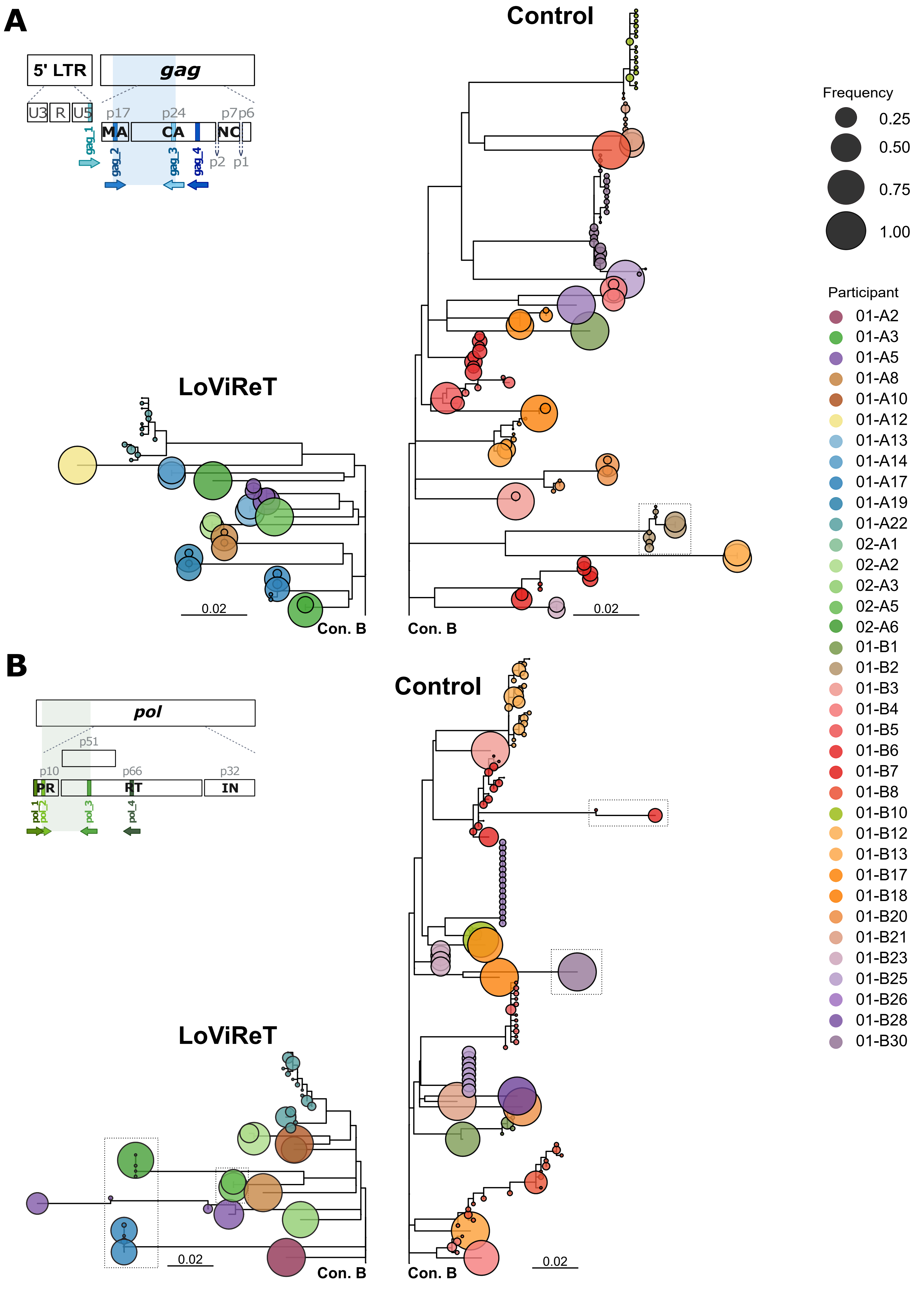

Supplement: Supplementary file 1 [file DataSheet1.pdf]
